# Uncovering novel KCC2 regulatory motifs through a comprehensive transposon-based mutant library

**DOI:** 10.3389/fnmol.2024.1505722

**Published:** 2025-01-15

**Authors:** Pavel Uvarov, Satoshi Fudo, Cem Karakus, Andrey Golubtsov, Federico Rotondo, Tatiana Sukhanova, Shetal Soni, Coralie Di Scala, Tommi Kajander, Claudio Rivera, Anastasia Ludwig

**Affiliations:** ^1^Neuroscience Center, HiLIFE, University of Helsinki, Helsinki, Finland; ^2^Institute of Biotechnology, HiLIFE, University of Helsinki, Helsinki, Finland; ^3^INSERM, INMED, Aix Marseille University, Marseille, France

**Keywords:** KCC2, SLC12A5, GABA, chloride homeostasis, Mu transposon mutagenesis, KCC2-CTD mutations

## Abstract

**Introduction:**

The neuron-specific K-Cl cotransporter KCC2 maintains low intracellular chloride levels, which are crucial for fast GABAergic and glycinergic neurotransmission. KCC2 also plays a pivotal role in the development of excitatory glutamatergic neurotransmission by promoting dendritic spine maturation. The cytoplasmic C-terminal domain (KCC2-CTD) plays a critical regulatory role in the molecular mechanisms controlling the cotransporter activity through dimerization, phosphorylation, and protein interaction.

**Methods:**

To identify novel CTD regulatory motifs, we used the Mu transposon-based mutagenesis system to generate a library of KCC2 mutants with 5 amino acid insertions randomly distributed within the KCC2-CTD. We determined the insertion positions in 288 mutants by restriction analysis and selected clones with a single insertion site outside known KCC2 regulatory motifs. We analyzed the subcellular distribution of KCC2-CTD mutants in cultured cortical neurons using immunocytochemistry and selected ten mutants with ectopic expression patterns for detailed characterization.

**Results:**

A fluorescent Cl^−^-transport assay in HEK293 cells revealed mutants with both reduced and enhanced Cl^−^-extrusion activity, which overall correlated with their glycosylation patterns. Live-cell immunostaining analysis of plasma membrane expression of KCC2-CTD mutants in cultured cortical neurons corroborated the glycosylation data. Furthermore, the somatodendritic chloride gradient in neurons transfected with the KCC2-CTD mutants correlated with their Cl^−^-extrusion activity in HEK293 cells. Gain- and loss-of-function mutant positions were analyzed using available KCC2 cryo-EM structures.

**Discussion:**

Two groups of mutants were identified based on 3D structural analysis. The first group, located near the interface of transmembrane and cytoplasmic domains, may affect interactions with the N-terminal inhibitory peptide regulating KCC2 activity. The second group, situated on the external surface of the cytoplasmic domain, may disrupt interactions with regulatory proteins. Analyzing CTD mutations that modulate KCC2 activity enhances our understanding of its function and is essential for developing novel anti-seizure therapies.

## 1 Introduction

Neuron-specific K-Cl cotransporter KCC2 maintains low intracellular Cl^−^ concentration ([Cl^−^]_i_) in neurons, rendering GABA_A_ responses hyperpolarizing (Hübner et al., 2001; Rivera et al., 1999). KCC2 malfunction leads to compromised GABAergic inhibition, which may contribute to the onset of epileptic seizures (Barmashenko et al., 2011; Deeb et al., 2013; Pathak et al., 2007). Numerous mutations in the human KCC2 protein are associated with the development of epilepsy (Järvelä et al., 2024; Kahle et al., 2014; Puskarjov et al., 2014; Saito et al., 2017; Saitsu et al., 2016; Stödberg et al., 2015). Additionally, conditions such as brain tumors, ischemic stroke, and brain injuries are known to downregulate KCC2 expression, thereby increasing the likelihood of epileptic seizures (Boulenguez et al., 2010; Conti et al., 2011; Jarolimek et al., 1999; Khirug et al., 2005; Pallud et al., 2014; Sawant-Pokam et al., 2020). Preclinical research suggests that augmenting KCC2 activity could suppress seizures, highlighting the KCC2 role in modulating neuronal excitability under normal and epileptogenic conditions (Jarvis et al., 2023; Lee et al., 2021; Moore et al., 2018).

KCC2 C-terminal domain (CTD) is crucial for regulating KCC2 functionality. It harbors multiple regulatory phosphorylation sites, including Y903, T906, S932, T934, S937, S940, T1007, T1009, S1034, and Y1087, which modulate activity and membrane trafficking of the cotransporter (Cordshagen et al., 2018; Lee et al., 2007; Rinehart et al., 2009; Weber et al., 2014). In addition, the KCC2-CTD bears a calpain cleavage site that mediates activity-dependent degradation of KCC2 (Puskarjov et al., 2012; Wan et al., 2018; Zhou et al., 2012) and a non-canonical dileucine motif that controls KCC2 endocytosis (Zhao et al., 2008). In addition, a short CTD sequence called ISO domain mediates constitutive activity of KCC2 under isotonic conditions (Acton et al., 2012; Mercado et al., 2006). Finally, actin cytoskeleton regulatory proteins such as 4.1 N and β-PIX bind to the KCC2-CTD, and through this interaction, influence the shape of dendritic spines and the plasticity of glutamatergic synapses (Li et al., 2007; Llano et al., 2015).

In this study, we report the generation and analysis of a mutant library targeting KCC2-CTD. We utilized the Mu transposon-based mutagenesis system to create 288 mutants with 5 amino acid random insertion sites within KCC2-CTD. Based on immunocytochemical analysis, we selected ten mutants displaying expression patterns distinct from the wild-type (WT) KCC2. Among selected clones, a fluorescent Cl^−^-transport assay conducted in HEK293 cells revealed mutants exhibiting both reduced and enhanced Cl^−^-extrusion activity. The somatodendritic chloride gradient, measured using a patch clamp in cultured neurons transfected with the KCC2-CTD mutants, supported the results of the Cl^−^-transport assay in HEK293. Notably, high KCC2 membrane expression and mature glycosylation pattern of certain mutants did not necessarily guarantee their high K-Cl cotransport activity.

Given the diverse regulatory functions of CTD, our insertion CTD library promotes identification of novel motifs modulating various aspects of KCC2 activity. Through systematic analysis of the positions of the CTD mutations, we can gain valuable insights into the mechanisms governing KCC2 regulation and potentially discover new targets for therapeutic intervention in neurological disorders.

## 2 Materials and methods

### 2.1 DNA constructs

As a template for the Entranceposon (M1-Kan) integration, we used the rat KCC2b ORF sequence containing one copy of FLAG-tag (DYKDDDDK) in the large 3rd extracellular loop and controlled by the CMV promoter (pCMV-KCC2_FLAG-out) (Pressey et al., 2017). The extracellular FLAG-tag was inserted into the low-conserved region of the 3rd extracellular loop between amino acid positions 383 and 384 of the rat KCC2b. The insertion site is located in the loop away from the ECD secondary structures and from the previously described glycosylation sites. KCC2_FLAG-out construct was previously characterized (Pressey et al., 2017) and found to be fully functional. pCAG-mGFP was a kind gift from Dr. Connie Cepko (Addgene plasmid #14757). pcDNA3.1(−) was used for mock transfections.

### 2.2 KCC2-CTD mutant library

The mutant library was generated using the commercially available transposition kit (Mutation Generation System Kit, catalog number F701, ThermoFisher Scientific) according to the manufacturer’s instructions. The Entranceposon (M1-Kan) integration reaction was optimized to ensure that most of the mutants contained only one integration site. Kanamycin resistance gene within the transposon cassette was used to select the mutant clones. To restrict the mutagenesis exclusively to the distal C-terminal regulatory part (809–1,116 aa) of the large KCC2-CTD (635–1,116 aa), the mutant DNA plasmid library was cut at the unique *Bst*EII and *Afl*II restriction sites embracing the indicated regulatory region and ~300 bp of the proximal 3′ untranslated region (3’UTR). The *Bst*EII-*Afl*II digestion products were gel-purified and ligated into the original unmutated KCC2 cDNA construct precut similarly at *Bst*EII and *Afl*II sites. As a result, the final KCC2-CTD library contained mutations exclusively within the indicated KCC2b cDNA region. The transposon insertion cassette carrying the Kan-resistance gene, flanked by two *Not*I sites, was removed by *Not*I digestion and sticky-end re-ligation, thus leaving 15 bp fragments containing a single *Not*I site. High-efficiency NEB 5α competent cells (New England Biolabs) were transformed by the resultant KCC2-CTD library, and 288 bacterial colonies (3× 96-well plates) were subjected to subsequent analysis.

### 2.3 Immunocytochemical analysis of KCC2 mutants in rat cultures

Rat cortical neurons were plated in a 96-well plate according to the protocol described previously (Ludwig et al., 2011). The cultures were transfected on day *in vitro* (DIV) 10 with a mixture of pCAG-mGFP and either pCMV-KCC2_FLAG-out or FLAG-KCC2-CTD mutants using Lipofectamine 2000 transfection reagent (ThermoFisher Scientific) according to the manufacturer’s protocol. Forty-eight hours after transfection, cultures were washed twice with 1x PBS and fixed in 4% PFA (50 μL/well) for 10 min, permeabilized with 0.1% Triton X-100 in 1x PBS for 5 min, and blocked in 10% normal donkey serum for 1 h. To study the intracellular distribution of the FLAG-KCC2-CTD mutants, the permeabilized cultures were incubated overnight with the mouse monoclonal anti-FLAG antibody (F3165, Sigma-Aldrich, 1:750). The next day, cultures were washed 3 times 20 min in 1x PBS and incubated with anti-mouse Cy5 (Jackson ImmunoResearch Laboratories, 1:400) secondary antibodies for 1 h at 37°C. DAPI was used to counterstain nuclei.

### 2.4 Immunocytochemical analysis of surface expression of KCC2 mutants in rat neurons

Rat cortical neurons were cultured on glass coverslips pretreated with poly-L-lysine as described previously (Ludwig et al., 2011). The cultures were transfected on DIV 6 with a mixture of mGFP and either wild-type or mutant KCC2_FLAG-out constructs using Lipofectamine 2000 transfection reagent (ThermoFisher Scientific) according to the manufacturer’s protocol. Two weeks after plating, transfected neurons were subjected to the surface immunolabeling protocol that comprises two steps. First step: Cultures were incubated with mouse anti-FLAG antibody (F3165, Sigma-Aldrich) diluted 1:200 in cell-culture medium for 30 min at 37°C, 5% CO_2_. The temperature and duration of the membrane KCC2 labeling step is the result of optimization by several groups working in the KCC2 field: 1 h at 37°C (Acton et al., 2012), 1.5 h at 37°C (Pressey et al., 2017), 2 h at 37°C (Friedel et al., 2017), and 1 h at 37°C (Järvelä et al., 2024). We found previously that lower temperatures resulted in a low labeling efficiency. After the incubation, cells were washed 3 times with ice-cold incubation buffer (NaCl, 127 mM; KCl, 3 mM, CaCl_2_, 2 mM; MgCl_2_, 1.3 mM; glucose, 20 mM; HEPES, 20 mM; pH 7.4) and incubated with anti-mouse Alexa Fluor 568 secondary antibody (Thermo Fisher Scientific) diluted 1:400 in the incubation buffer for 30 min at 13°C. This step reveals plasma membrane KCC2 immunoreactivity. Second step: Cells were washed 3 times with ice-cold incubation buffer and fixed with 4% PFA for 10 min at room temperature. After the fixation cells were washed 3 times with PBS, permeabilized with 0.1% TritonX-100 in PBS (TritonX/PBS), and the background signal was blocked with 10% goat serum in TritonX/PBS for 30 min at room temperature. After the blocking, cells were incubated with mouse anti-FLAG antibody (F3165, Sigma-Aldrich) diluted 1:400 in TritonX/PBS overnight at 4°C. Then cells were washed 3 times with TritonX/PBS and incubated with anti-mouse Alexa Fluor 647 secondary antibody (Thermo Fisher Scientific) diluted 1:400 in TritonX/PBS for 1 h at room temperature. After incubation with the secondary antibody, coverslips were washed once with TritonX/PBS, once with PBS, and once with distilled H_2_O, and mounted with Fluoromount-G (Southern Biotechnology). The second step reveals total KCC2 immunoreactivity.

### 2.5 Fluorescent Cl^−^ extrusion assay and analysis

For Cl^−^ extrusion assay HEK293T cells were cultured in Dulbecco’s modified Eagle’s medium supplemented with 10% fetal bovine serum and penicillin/streptomycin antibiotic mix. The cells were cotransfected with SuperClomeleon (Grimley et al., 2013) and KCC2-CTD mutants using Lipofectamine 2000 according to the manufacturer’s protocol. Cl^−^ efflux assay was performed 40–44 h after transfection using a protocol described previously (Friedel et al., 2013) with some modifications. Briefly, HEK293 cells were pre-incubated in the extracellular solution (ECS: 140 mM NaCl, 2.5 mM KCl, 10 mM HEPES, 20 mM D-glucose, 2 mM CaCl_2_, 2 mM MgCl_2_, pH 7.4, 300 mOsm). Next, cells were loaded with Cl^−^ by incubation for 10 min in 60 μL of the loading solution with high [K^+^]_o_ and high [Cl^−^]_o_ (75 K^+^, 65 mM NaCl, 75 mM KCl, 10 mM HEPES, 20 mM D-glucose, 2 mM CaCl_2_, 2 mM MgCl_2_, pH 7.4, 300 mOsm). The high [K^+^]_o_ rendered the membrane potential at the level sufficient for Cl^−^ loading into HEK293 cells via endogenous Cl^−^ channels and transporters. Previously we showed that raising the extracellular [K^+^]_o_ up to 50 mM results in efficient Cl^−^ loading even in the absence of GlyR-α1 glycine receptors and glycine (Ludwig et al., 2017). Subsequent addition of 300 μL of the K^+^-free solution (140 mM NaCl, 10 mM HEPES, 20 mM D-glucose, 2 mM CaCl_2_, 2 mM MgCl_2_, pH 7.4, 300 mOsm) was used to decrease [K^+^]_o_, thus initiating the KCC2-mediated Cl^−^ extrusion in the resultant extrusion solution (127.5 mM NaCl, 12.5 mM KCl, 10 mM HEPES, 20 mM D-glucose, 2 mM CaCl_2_, 2 mM MgCl_2_, pH 7.4, 300 mOsm). VU0463271 (VU) stock solution was prepared in DMSO (30 mM), stored at −20°C, and used within a week. All solutions containing working concentrations of VU (30 μM) were prepared freshly 1–2 h before the Cl^−^ efflux assay.

Cl^−^-sensitive (YFP) and Cl^−^-insensitive (Cerulean) components of SuperClomeleon fluorescence were detected sequentially using the microplate reader (FLUOstar Optima FL, BMG Labtech) equipped with the filter sets for YFP (excitation 500 nm, emission 560 nm) and Cerulean (excitation 450 nm, emission 480 nm). F_480_/F_560_ ratio, which directly correlates with the [Cl^−^]_i_, was recorded every 100 s during the loading (10 min) and extrusion (50 min) steps for VU+ and VU- wells corresponding to the same KCC2-CTD mutant. F_480_ and F_560_ background levels were measured in neighbor wells containing non-transfected HEK293 cells and were subtracted from the corresponding F_480_ and F_560_ values measured for the KCC2-CTD mutants. Analysis was performed using a custom-made Python script.

### 2.6 E_GABA_ gradient-based Cl^−^ extrusion assay

The assay of neuronal Cl^−^extrusion is based on imposing a somatic chloride load through a whole-cell patch-clamp electrode and measuring the somatodendritic gradient of the reversal potential of GABA_A_ receptor-mediated current responses (E_GABA_) induced along the dendrite by local iontophoretic application of GABA. Somatic recordings in immature cultured hippocampal neurons (DIV 3) were performed in standard extracellular solution at 34°C in the whole-cell voltage-clamp configuration using an EPC 10 patch-clamp amplifier (HEKA Elektronik, Inc., Germany). The composition of the extracellular solution was (in mM): 127 NaCl, 3 KCl, 2 CaCl_2_, 1.3 MgCl_2_, 10 D-glucose, and 20 HEPES, pH 7.4 was adjusted with NaOH. Patch pipettes were fabricated from borosilicate glass (Harward Apparatus, USA), and their resistances ranged from 6.5 to 8.5 MΩ. The composition of the patch pipette solution was (in mM): 29 KCl, 111 K-gluconate, 0.5 CaCl_2_, 2 NaOH, 10 D-glucose, 10 HEPES, and 2 Mg-ATP, 5 BAPTA, pH 7.3 was adjusted with KOH. For local iontophoretic application of GABA, brief 100 ms positive current pulses (50–70 nA) were delivered by a sharp micropipette (40–45 MΩ when filled with 250 mM GABA in 250 mM HCl). Iontophoretic GABA injections were given no more than once per minute. A constant negative current of −5 nA was applied to the micropipette to compensate for the passive leak of GABA. GABA was applied at the soma and the dendrite (∼30 μm from the soma) of a given neuron. Membrane potential was clamped at −60 mV. All membrane potential values were corrected for a liquid-junction potential of 14.16 mV. Before recording, the first 2 min were dedicated to loading the whole cell with the intracellular solution. The reversal potential of GABAergic currents (E_GABA_) was determined from the current–voltage (I–V) relation obtained using ramp voltage protocol. NKCC1 was blocked throughout the experiments with 10 μM bumetanide, action potentials with 1 μM TTX, and GABA_B_ receptors with 1 μM CGP 35348 (Abcam). Under these conditions, the somatodendritic gradient ΔE_GABA_ provides a quantitative estimate of the efficacy of KCC2-mediated Cl^−^ extrusion.

### 2.7 Glycosylation analysis of KCC2-CTD mutants in HEK293 cells

Except for deglycosylation experiments, samples were prepared as described before (Uvarov et al., 2009), with small modifications. Briefly, HEK293T cells cultured in Dulbecco’s modified Eagle’s medium supplemented with 10% fetal bovine serum were transfected with 1 μg of each plasmid carrying a KCC2 mutant gene using 3 μg of polyethylenimine (PEI) and incubated for 48 h. For SDS-PAGE analysis without deglycosylation treatment, the cell samples were homogenized on ice in lysis buffer (50 mM Tris–HCl, 150 mM NaCl, 1% Triton X-100, 0.5% deoxycholic acid, 0.1% SDS, pH 8.0) supplemented with the Pierce protease inhibitor mini tablets, EDTA-free (Thermo Fisher). For SDS-PAGE analysis with deglycosylation treatment, the cell samples were lysed on ice in TNE buffer (1% Nonidet P-40, 50 mM Tris–HCl, 140 mM NaCl, 5 mM EDTA, pH 8.0), supplemented with the Pierce protease inhibitor mini tablets, EDTA-free (ThermoFisher) and 100 μM iodoacetamide (Sigma). After centrifugation for 10 min (15,000 × *g*) at 4°C, protein concentrations of the supernatants were determined by using Pierce BCA protein assay kit (ThermoFisher).

The supernatants of HEK293 cell lysates containing KCC2 mutant proteins in TNE buffer were treated with PNGase F (New England Biolabs) and Endo H (New England Biolabs) according to manufacturer’s instructions with a slight modification. Briefly, each sample was mixed with Glycoprotein Denaturing Buffer and heated at 60°C for 20 min. For PNGase F reaction, the sample was then mixed with GlycoBuffer 2, Nonidet P-40 (final 1%), and PNGase F and incubated at 37°C for 1 h. For Endo H reaction, the sample was mixed with GlycoBuffer 3 and Endo H and incubated at 37°C for 1 h. For negative controls, a duplicate was made for each sample following the same procedure except that PNGase F or Endo H was not added to the duplicate. The resultant reactions were analyzed by SDS-PAGE.

### 2.8 SDS-PAGE and Western blotting

Each sample containing 30 μg protein was mixed with 5× Laemmli loading buffer (300 mM Tris–HCl, 10% SDS, 50% glycerol, 0.05% bromophenol blue, pH 6.8) and DTT (final 70 mM). The lysates were not heated before loading on the SDS-PAGE. The protein samples were separated using 7.5% Mini-PROTEAN TGX precast protein gels (Bio-Rad) at 150 V for 60 min and then transferred onto PVDF membrane with Trans-Blot Turbo Transfer System (Bio-Rad).

Blots were blocked with 5% skim milk in Tris-buffered saline with 0.1% Tween 20 and probed with rabbit anti-KCC2 antibody (Millipore, 1:2500 dilution) and subsequently with horseradish peroxidase-conjugated goat anti-mouse IgG antibody (Santa Cruz). Blots were developed with Amersham ECL Prime Western Blotting reagents (Merck), visualized with ChemiDoc XRS+ (Bio-Rad), and analyzed with Image Lab software (Bio-Rad). All measurements were within the linear range of camera sensitivity.

## 3 Results

### 3.1 Generation of the KCC2-CTD mutant library and mapping the mutations

**Figure 1:**
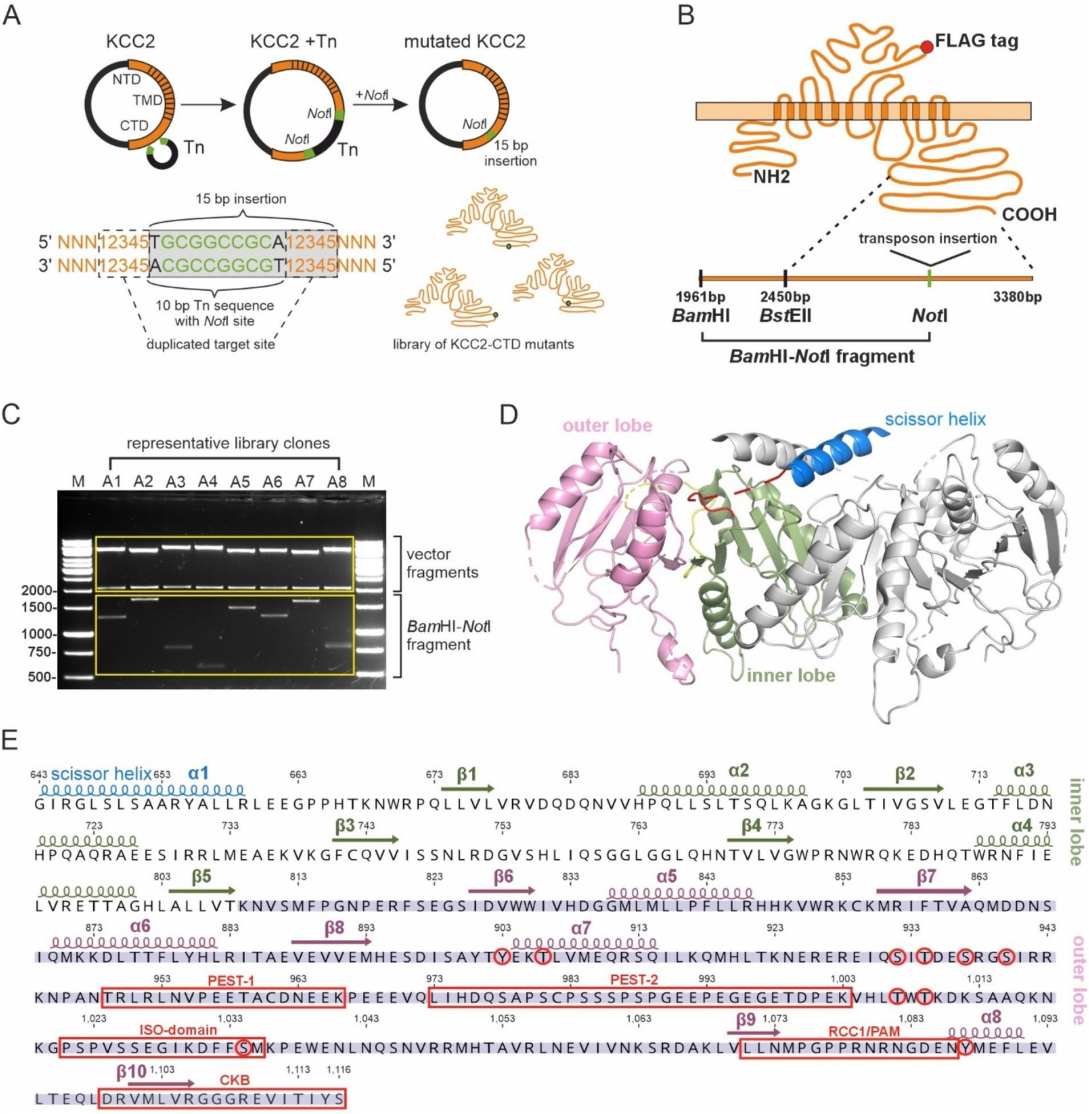
Generation of the KCC2-CTD mutant library. **(A)** A schematic drawing of the Entranceposon mutagenesis approach applied to KCC2-CTD. A single *Not*I restriction site, which remained after the removal of the transposon DNA from KCC2 cDNA, was used to identify insertion sites. **(B)** A schematic drawing of the KCC2 protein with the FLAG-tag, along with a schematic of the C-terminus depicting restriction sites used for insertion mapping. **(C)** A representative agarose gel image showing DNA fragments following digestion with *Bam*HI and *Not*I. The size of the *Bam*HI-*Not*I fragment was used to determine the mutation location. **(D)** Cryo-EM structure (PDB:7D14) of the mouse KCC2 C-terminal domain. The KCC2 dimer is depicted with the scissor helix (blue), inner lobe (green), and outer lobe (pink) of one KCC2 subunit, while the corresponding parts of the second subunit are shown in gray. **(E)** The mouse KCC2b-CTD amino acid sequence, with the corresponding secondary structure elements (PDB:7D14) colored as in **(D)**, constituting the inner and outer lobes. Previously characterized phosphorylation sites and sites of KCC2-interacting proteins are shown in red.

To identify regulatory motifs in the KCC2 C-terminal domain (CTD), we generated a library of the full-length KCC2b mutants containing bacteriophage Mu-mediated random insertions of 15 bp nucleotide sequence into the KCC2b cDNA part encoding the distal C-terminal domain between amino acids 809 and 1,116 (Figure 1A). This mutagenesis strategy resulted in 5-aa in-frame insertions within the distal CTD. The exact amino acid sequence of the insertions depended on the nucleotide sequence surrounding the insertion site. When inserted into the open reading frame of a protein, the transposon sequence ensured the absence of premature stop codons within the mutated region. To map the positions of the insertions in the KCC2-CTD library, we purified 288 plasmids corresponding to the individual mutants and cut them with restriction enzymes *Bam*HI (located 489 bp upstream of the mutated CTD region) and *Not*I (at the insertion site) (Figure 1B). The *Bam*HI - *Not*I restriction products ranged between ~500 and ~1700 bp (Figure 1C), with a total of 288 mutations spread across the mutated region, including 221 located between 809 and 1,116 aa of KCC2-CTD and 67 in the 300 bp fragment of the KCC2 proximal 3’UTR. In agreement with the experimental design, the insertions were outside the inner CTD lobe (643–809 aa), which comprises the previously described (Chi G. et al., 2021) scissor helix and β1-β5 strands required for KCC2 dimerization (Figures 1D,E).

### 3.2 Somatodendritic distribution of the KCC2-CTD mutants in neurons

Previous studies have shown that KCC2 expression and lateral diffusion in the plasma membrane strongly depend on the CTD phosphorylation (Conway et al., 2017; Friedel et al., 2015; Heubl et al., 2017; Inoue et al., 2012; Lee et al., 2007) and interaction with regulatory proteins (Chamma et al., 2013; Li et al., 2007; Llano et al., 2015). Since most 5-aa insertions were located in the CTD outer lobe, we expected their possible effect on the KCC2 somatodendritic distribution. Thus, dissociated cortical cultures were transiently transfected with 96 randomly selected KCC2 mutants and analyzed using antibodies recognizing the FLAG-tag placed in the 3rd extracellular loop, outside the previously characterized glycosylation sites and secondary structure elements. The transfection mixture also contained GFP, enabling the identification of the transfected neurons and the analysis of the effect of KCC2-CTD mutants on the morphology of dendritic trees in transfected neurons. For each mutant, FLAG-immunoreactivity (FLAG-ir) in the transfected neurons was examined considering several parameters: (1) total signal intensity, (2) somatic distribution, and (3) distribution in proximal and distal dendrites.

**Figure 2:**
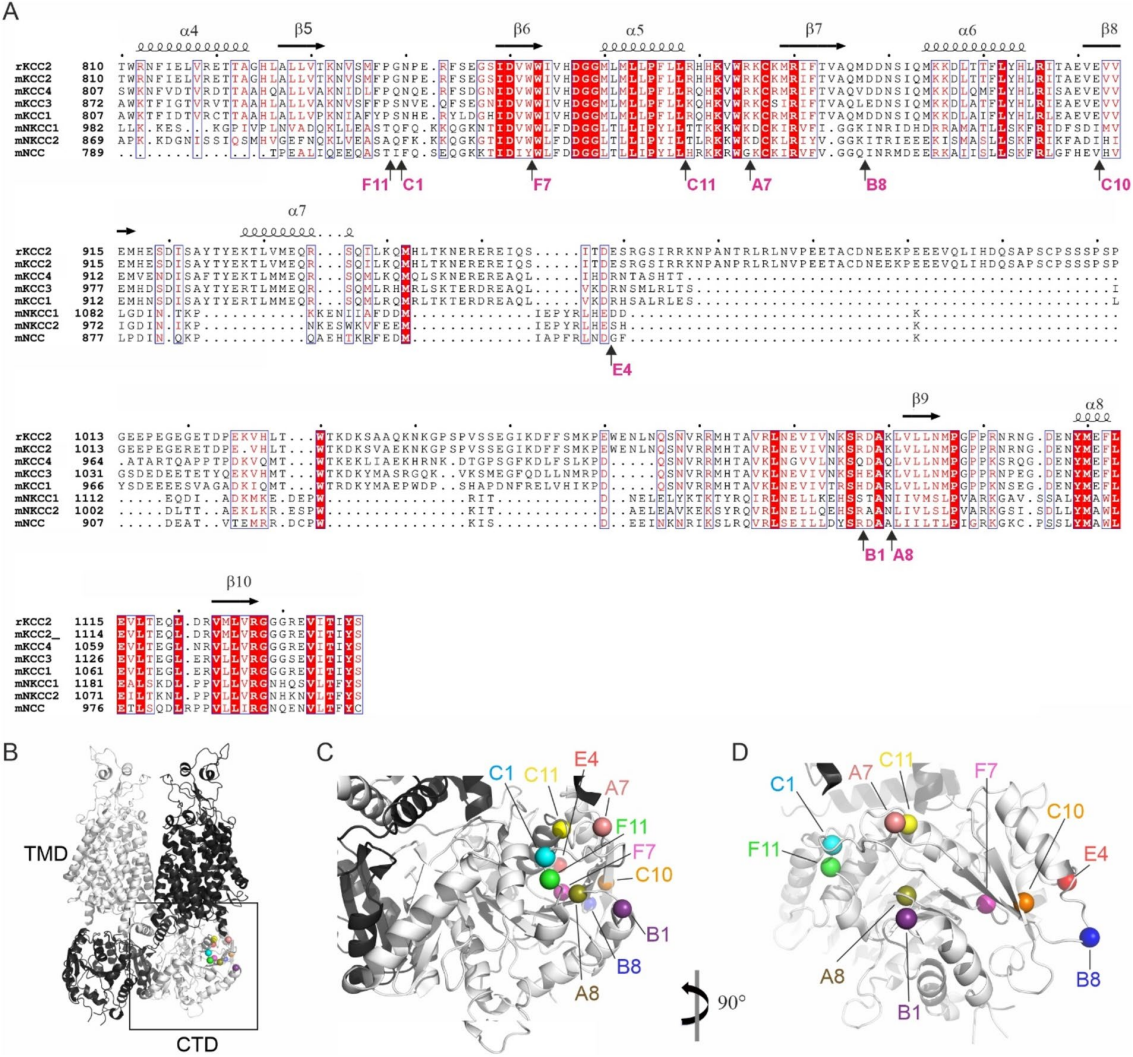
Location of the ten KCC2-CTD mutants chosen for detailed characterization. **(A)** Protein sequence alignment of the distal CTD region of mouse SLC12 family members and the rat KCC2, which served as the basis for mutagenesis in this study. The selected mutations are located in both the poorly conserved, unstructured fragments and in the highly conserved structural elements, including *α*-helices and β-strands. Mutation sites are indicated by arrows, along with the name of the insertion mutants. Secondary structure elements are annotated based on the mouse KCC2 cryo-EM structure PDB: 7D14 (Zhang et al., 2021). **(B)** Overall 3D view of the KCC2 dimer represented in white and black for each monomer. CTD, cytoplasmic domain; TMD, transmembrane domain (PDB:6 M23, Chi X. et al., 2021). **(C,D)** Zoomed-in views of the boxed region (CTD) in panel **(B)** at different angles, as indicated, showing the insertion sites in the mutant library as spheres.

Out of 96 analyzed mutants, 14 demonstrated ectopic FLAG-ir and/or GFP expression patterns and were sequenced to identify positions of the 5-aa insertion. Ten sequenced mutants (Table 1) with unique insertion sites located outside the previously characterized KCC2 regulatory motifs PEST-1, PEST-2, ISO-domain, CKB, RCC1/PAM (Supplementary Figure 1) were chosen for detailed functional characterization. The selected mutations were found within both the unstructured linker-type sequences and the highly conserved *α*-helices and *β*-strands (Figure 2A). Except for A8 and B1, all selected mutants demonstrated moderate to high levels of the somatic FLAG-ir (Supplementary Figure 2). Dendritic levels of FLAG-ir varied significantly, being highest for B8 and C1, but lowest for A7, A8, and C10 mutants. Somatic morphology was affected in neurons expressing A7, B1, C10, and C11 mutants, whereas dendritic morphology was distorted in nearly all mutants. Alterations included lamellipodia formation, malformed, short, or thin dendrites, as well as focal swelling of dendrites, known as dendritic blebbing.

**Table 1:** Summary for the immunocytochemical analysis of the selected KCC2-CTD mutants.

	FLAG-immunoreactivity			Morphology		
CTD-mutant	Cell body	Primary dendrites	Other dendrites	Dendritic tree	Cell body	Notes
A7	++	+/−	−	Lamellipodia	Distorted shape	Somatic expression
A8	+	+/−	−	Short distorted dendrites, lamellipodia		
B1	+/−	−	−	Distorted shape, beading	Distorted shape	
B8	+++	+++	+++	Distorted, beading, few dendrites per cell		Strong FLAG-ir in dendritic spines
C1	+++	+++	+++	Distorted, beading, few dendrites per cell		Strong FLAG-ir in dendritic spines
C10	++	+	−	Distorted dendrites, lamellipodia	Distorted shape	
C11	++	++	+	Short distorted dendrites, beading, lamellipodia	Distorted shape	
E4	+++	++	++	Short dendrites, beading, lamellipodia		
F7	++	++	+	Short dendrites, many spines/filopodia		Strong FLAG-ir in dendritic spines
F11	+++	++	+	Multiple thin dendrites		

Expression level: +/− poor, + weak, ++ moderate, +++ strong.

The observed morphological changes in soma and dendrites could result from the altered intracellular ion balance due to the altered K-Cl cotransporter activity (Weilinger et al., 2022) or from the malformed cytoskeleton caused by the impaired KCC2 interaction with the previously identified actin-regulating partners, e.g., 4.1 N and β-PIX (Li et al., 2007; Llano et al., 2015). The position of the KCC2 mutants in the outer lobe suggests that both scenarios are possible (Figures 2B–D). One group of the mutants (A7, C1, C11, and F11) is located near the interface between the transmembrane domain (TMD) and CTD, known to contribute to the interaction with the KCC2 N-terminal inhibitory peptide that directly regulates KCC2 efflux activity (Zhang et al., 2021). Mutants from another group (A8, B1, B8, C10, and E4) are located on the external side surface of the KCC2-CTD, potentially disrupting the interaction with regulatory proteins. Therefore, in the next step, we measured the K-Cl cotransporter activity of the selected mutants.

### 3.3 Cl^−^ efflux activity of the KCC2-CTD mutants in HEK293 cells

**Figure 3:**
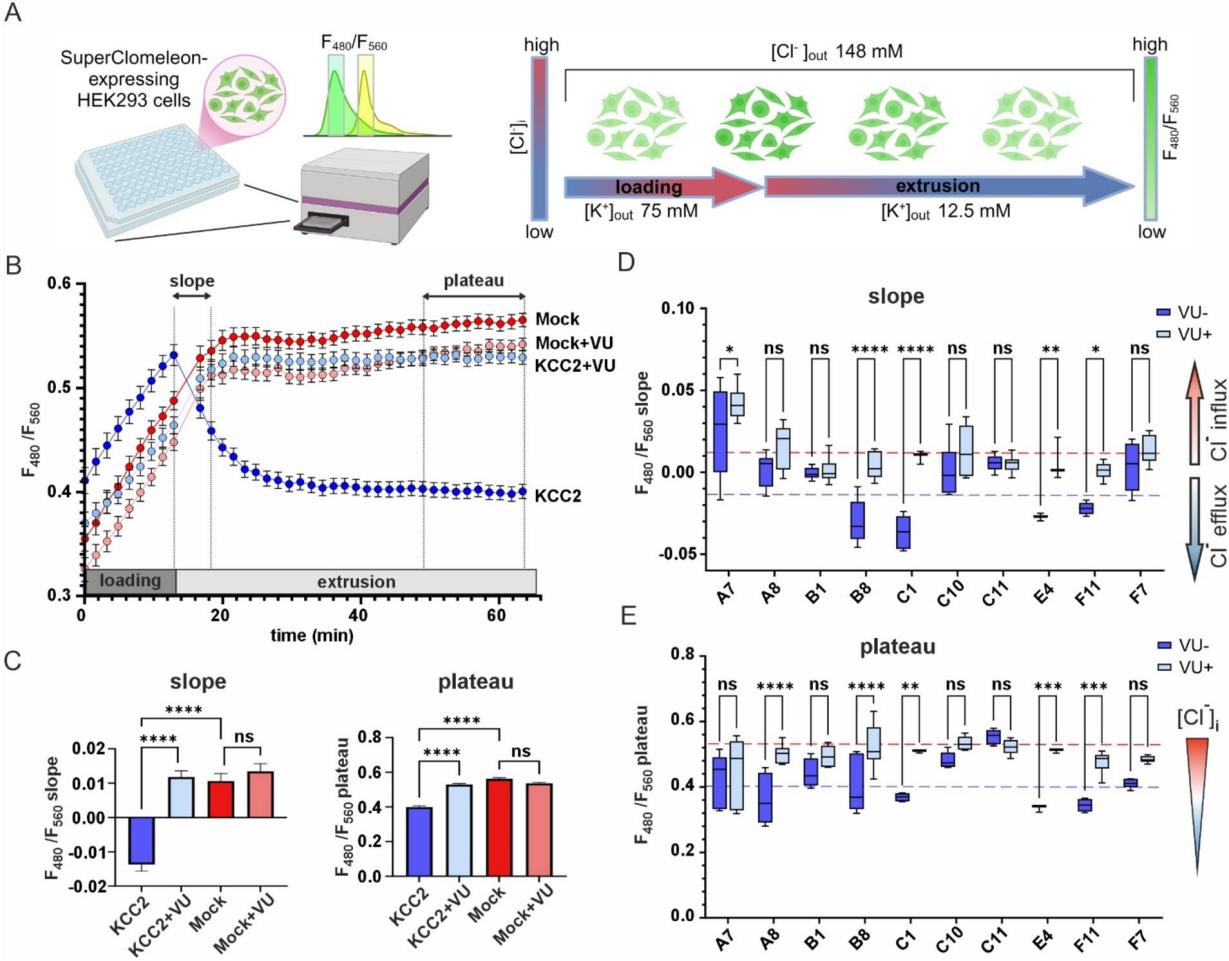
K-Cl cotransporter activity of the KCC2 mutants. **(A)** A schematic drawing illustrating the design of the chloride efflux assay. **(B)** F_480_/F_560_ ratio over time for pcDNA3- (Mock) and KCC2- transfected cells in the absence or presence of VU. The time intervals used for calculating the slope and the plateau values are indicated on the top part of the graph by dashed lines and double-headed arrows. **(C)** F_480_/F_560_ slope (left panel) and plateau (right panel) values for pcDNA3- and KCC2-transfected cells in the absence or presence of VU. Bars represent the mean; error bars are SEM. Kruskal-Wallis test with Dunn’s post-hoc test was used to compare the constructs to each other, ****p < 0.0001, ns – not significant. **(D)** F_480_/F_560_ slope data for ten KCC2 mutants. Each box represents 25th to 75th percentiles with the median marked by a line; whiskers denote the minimum to the maximum values. Two-way ANOVA with Bonferroni post-hoc test was used to compare mutants under +/− VU conditions. The plateau levels corresponding to the WT KCC2 and Mock are indicated by red and blue dashed lines, respectively. *p < 0.05, **p < 0.01, ***p < 0.001, ****p < 0.0001, ns – not significant. **(E)** F_480_/F_560_ plateau data for ten KCC2 mutants. Data presentation and statistical tests are the same as in **(D)**.

To assess the K-Cl cotransport activity of the KCC2 mutants, we utilized the Cl^−^ – sensitive fluorescent reporter SuperClomeleon (Grimley et al., 2013). The standard assay began by perfusing the HEK293 cells with a high-K^+^ solution to promote an increase in intracellular [Cl^−^]_i_ (loading step), followed by a low-K^+^ solution to induce KCC2-driven Cl^−^ efflux, thereby reducing intracellular [Cl^−^]_i_ (extrusion step) (Figure 3A). The rate of [Cl ^−^]_i_ decline and the steady-state plateau level of [Cl^−^]_i_ achieved at the end of the extrusion step were assessed by monitoring the emitted SuperClomeleon fluorescence at 480 nm ([Cl^−^]_i_-insensitive Cerulean fluorescence) and 560 nm ([Cl^−^]_i_-sensitive YFP fluorescence). The F_480_/F_560_ ratio was used as an indicator of the [Cl^−^]_i_, with a lower ratio corresponding to lower [Cl^−^]_i_ levels.

HEK293 cells transfected with the nonmutant KCC2 construct showed a steep increase of F_480_/F_560_ ratio from 0.41 ± 0.01 to 0.53 ± 0.01 during the loading step, a robust drop to 0.46 ± 0.01 during first 5 min of the extrusion step and continued to slowly decrease until reaching a plateau level of 0.40 ± 0.06 during the last 10 min of the extrusion step (Figure 3B, dark blue). KCC2-specific inhibitor VU0463271 (VU) completely abolished the F_480_/F_560_ ratio decrease during the extrusion step (Figure 3B, light blue). HEK293 cells lacking KCC2 (mock-transfected), with or without VU (Figure 3B, dark and light red curves, respectively), did not reveal any drop in F_480_/F_560_ ratio. These data indicate that the slope of the F_480_/F_560_ curve during the first 5 min and the F_480_/F_560_ ratio averaged during the last 10 min of the extrusion step can both be used as an indicator of KCC2-mediated K-Cl transport activity (Figure 3C).

Next, we measured the F_480_/F_560_ slopes and the corresponding plateau levels for the ten selected KCC2-CTD mutants in the absence and presence of VU (Figures 3D,E and Supplementary Figure 3). Four mutants: B1, C10, C11, and F7, showed no significant difference between +/− VU conditions for the slope and the plateau, indicating that the K-Cl cotransporter activity in these mutants was impaired. All these mutations were localized within or adjacent to conserved secondary structural elements (see Figure 2 and Table 2): C10 in β8-strand, C11 in α5-helix, F7 in β6-strand, B1 3-aa upstream of the β9-strand. This suggests that these mutations may have significantly affected CTD structure.

**Table 2:** VU-sensitive Cl^−^ extrusion activities of the KCC2 mutants, along with positions of mutations, insertion sequences, and structural elements at the mutation positions according to PDB: 7D14.

Mutant	Position (aa)	Insert	Structural element	VU-sensitive Cl^−^ flux (KCC2 = 100%)	
				Slope	Plateau
F11	816	VRPHP		90 ± 7	102 ± 5
C1	817	CGRTG		232 ± 7	104 ± 6
F7	830	LRPQW	β6-strand	32 ± 27	64 ± 4
C11	846	CGRIL	α5-helix	−15 ± 9	−4 ± 6
A7	853	CGRRR		60 ± 39	35 ± 16
B8	865	RPQQM		137 ± 20	96 ± 17
C10	890	CGRME	β8-strand	39 ± 30	50 ± 7
E4	936	CGRTD		68 ± 31	65 ± 29
B1	1,066	CGRTR	3-aa upstream β9	-4 ± 5	50 ± 9
A8	1,069	CGRTK	β9-strand	56 ± 15	105 ± 15
Mock				0 ± 10	0 ± 4

A8 mutant did not exhibit significant VU-dependent Cl^−^ flux based on the slope measurements but reached the final plateau level comparable to WT KCC2 (Figure 3E, blue dashed line). This indicates the low transport activity of the A8 mutant, which is nevertheless sufficient for rendering the low steady-state [Cl^−^]_i_ in the absence of substantial Cl^−^ leakage. Like other nonfunctional mutants mentioned above, A8 mutation is also located within the conserved secondary structural element (β9-strand, see Table 2).

In contrast to other KCC2 mutants, A7 showed a significant positive F_480_/F_560_ slope, indicating that [Cl^−^]_i_ level rapidly increased at the beginning of the extrusion step (Figure 3D). The addition of VU to the extrusion solution further increased this slope. Since the extrusion solution has low [K^+^] and the driving force for K^+^ is directed outward, Cl^−^ influx under these conditions implies that A7 mutant has lost either the coupling between K^+^ and Cl^−^ ions or the ion selectivity. The remaining mutants (B8, C1, E4, and F11) exhibited significant differences between +/− VU conditions for both the slope and the plateau measurements, indicating that these mutants possess Cl^−^-extruding activity. Notably, all four mutations are located in poorly conserved CTD regions (Figure 2) outside known secondary structural motifs (see Table 2). Also, three out of four mutants (B8, C1, and F11) are located outside previously characterized KCC2-regulatory sites, while one (E4) is next to the previously characterized S940 phosphorylation site (Supplementary Figure 1). Interestingly, B8 and C1 demonstrated prominent negative slopes, indicating faster Cl^−^ extrusion kinetics compared to WT KCC2 (blue dashed line in Figure 3D). To sum up, mutating the distal KCC2-CTD region modulates the K-Cl cotransporter function both negatively and, what is important, positively.

### 3.4 KCC2-CTD mutants reveal different glycosylation patterns in the HEK293 cells

The observed effect of the KCC2-CTD mutations on K-Cl cotransporter function may result from changes in Cl^−^ extrusion kinetics or alterations in KCC2 abundance in the plasma membrane. Since protein trafficking to the plasma membrane is intimately linked to its glycosylation status, we next analyzed the glycosylation pattern of KCC2 mutants. Two protein bands with molecular weights close to the predicted non-glycosylated KCC2b polypeptide (123.6-kDa) have been previously reported in KCC2-overexpressing HEK293 lysates by SDS-PAGE (Agez et al., 2017; Blaesse et al., 2006; Williams et al., 1999), implying at least two glycosylation states (Payne et al., 1996; Kok et al., 2024).

**Figure 4:**
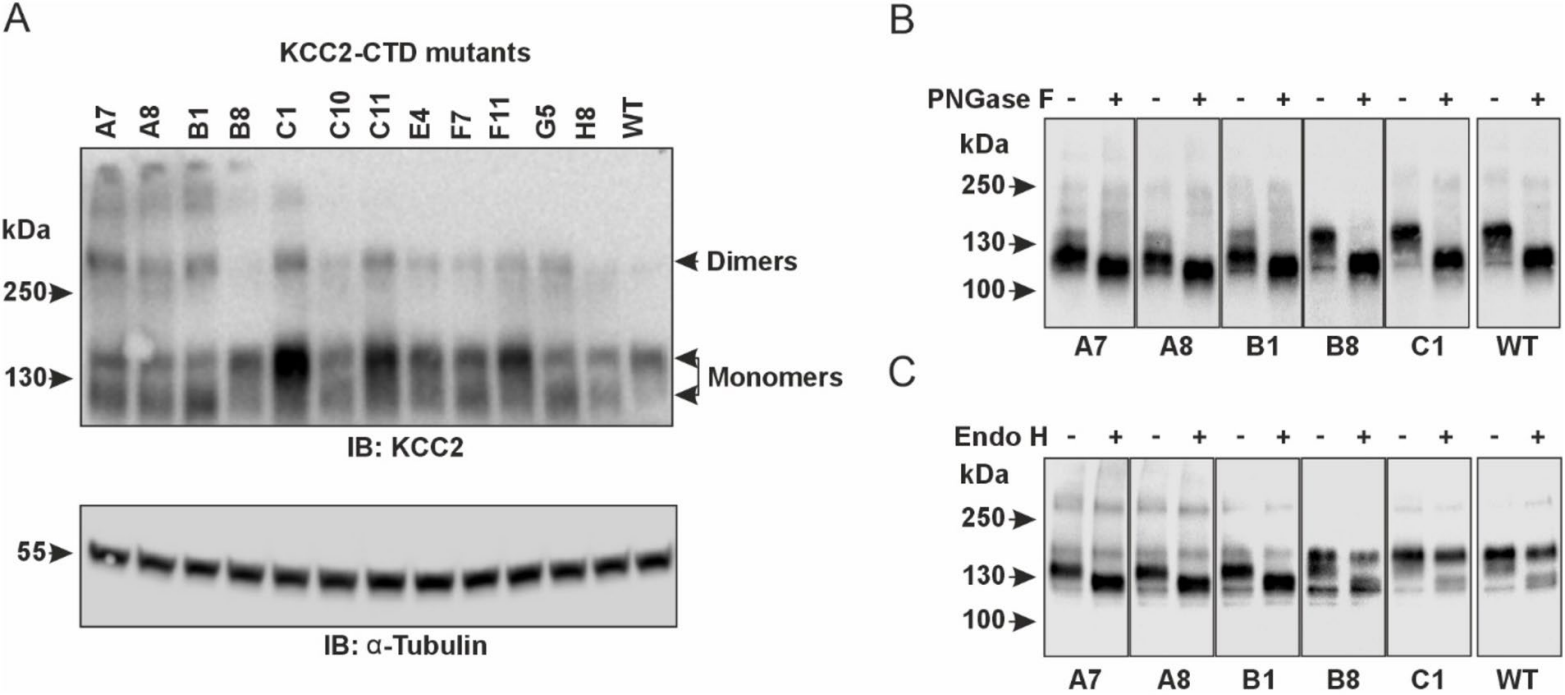
Glycosylation patterns of the KCC2-CTD mutants. **(A)** Top panel: Western blot analysis of KCC2 polypeptides in the total protein lysates of HEK293 cells expressing KCC2-CTD mutants, using a KCC2 antibody recognizing the C-terminal epitope. Two bands around 130-kDa corresponding to the putative glycosylated forms of monomeric KCC2 are observed. Bottom panel: To ensure equal amounts of total proteins loaded on SDS–PAGE, blots were analyzed with the antibody recognizing a-Tubulin. **(B)** PNGase F treatment removes all N-linked oligosaccharides from the KCC2 polypeptides, thus shifting the KCC2 bands to their predicted unglycosylated molecular weight of 123.6-kDa. **(C)** Endo H treatment shifts down the 125-kDa band corresponding to the A7, A8, and B1 KCC2 mutants, which contain mainly ER-added high-mannose glycans but leaves intact the 140-kDa KCC2 band corresponding to the B8 and C1 mutants, which contain mainly Golgi-added complex glycans.

In agreement with the previous studies, our western blot data revealed two monomeric KCC2 bands close to ~130-kDa in total protein lysates of HEK293 cells expressing CTD mutants (Figure 4A). The upper one (~140-kDa), presumably corresponding to the fully glycosylated KCC2 form, was prevalent in the cells expressing the WT KCC2 as well as B8, C1, C11, E4, and F11 CTD mutants. The remaining mutants (A7, A8, B1, C10, F7) showed comparable intensity for the upper (~140-kDa) and the lower (~125-kDa) KCC2 bands. To test whether N-glycosylation causes the observed difference in molecular weights of the two KCC2 monomeric bands, HEK293 lysates were treated with peptide-N-glycosidase F (PNGase F), known to remove most of the N-linked oligosaccharides (Tarentino and Plummer, 1994). Indeed, PNGase F treatment shifted both the 140-kDa and the 125-kDa bands for WT KCC2 and all CTD mutants down to the predicted 123.6-kDa molecular weight for the non-glycosylated KCC2 (Figure 4B). To examine whether the upper 140-kDa band corresponds to the mature Golgi-type glycosylation pattern rather than to the immature ER-type, HEK293 protein lysates were treated with the Endoglycosidase H (Endo H). Endo H cleaves oligosaccharides added to the proteins in the ER but not those processed in the Golgi, and thus is often used for assessing the extent of protein trafficking from ER toward the plasma membrane. As expected, the upper 140-kDa band, but not the lower 125-kDa, was resistant to the Endo H treatment (Figure 4C), thus pointing at its mature N-glycosylation pattern acquired in the Golgi by the B8, C1, C11, E4, and F11 CTD mutants.

Based on glycosylation and Cl^−^ efflux data, the analyzed KCC2-CTD mutants could be divided into two groups. The first group includes the B8, C1, E4, and F11 mutants, which exhibited both high Cl^−^ efflux activity and a predominant Golgi glycosylation pattern (140-kDa band). The second group comprises the A7, A8, B1, C10, and F7 mutants, which showed no VU-sensitive Cl^−^ efflux activity and displayed an immature ER-like glycosylation pattern – indicative of low plasma membrane expression. The immature glycosylation pattern in this group could be attributed to impaired KCC2 conformation, leading to a failure to exit the ER, which in turn prevents KCC2 trafficking to the Golgi and the plasma membrane. In most cases, retention of KCC2 mutants in the ER may explain the low Cl^−^ efflux activity. Interestingly, the C11 mutant lacked VU-sensitive Cl^−^ efflux activity but exhibited a highly mature glycosylation pattern, suggesting that the main effect of the mutation was impairment of the transporter’s kinetic activity rather than its trafficking to the plasma membrane.

### 3.5 The abundance of the KCC2-CTD mutants in the neuronal plasma membrane correlates with their glycosylation patterns in HEK293 cells

**Figure 5:**
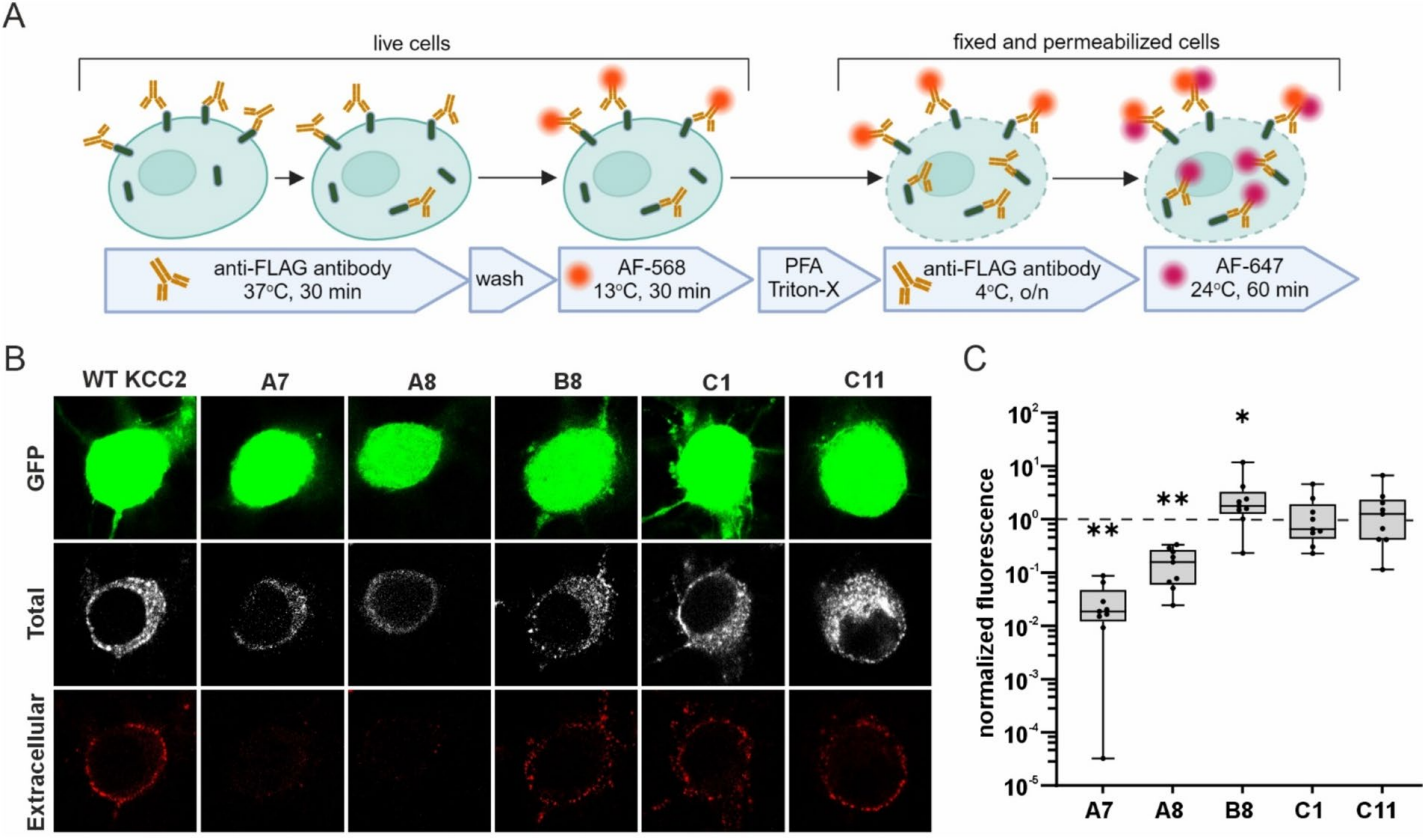
Surface expression of the KCC2-CTD mutants in cortical neurons. **(A)** A schematic drawing illustrating the live- cell labeling protocol. **(B)** Representative images of cortical neurons expressing WT KCC2 and selected KCC2-CTD mutants. Panels show GFP expression, total FLAG-ir (“Total”), and extracellular FLAG-ir (“Extracellular”). **(C)** Surface expression of the KCC2-CTD mutants normalized to that of WT KCC2 (indicated by the dashed line on the plot). Each box represents 25th to 75th percentiles with the median marked by a line; whiskers denote the minimum to the maximum values; one sample Wilcoxon test, *p < 0.05, **p < 0.01.

To test whether the glycosylation patterns observed in HEK293 cells correlate with the abundance of the KCC2-CTD mutants in the neuronal plasma membrane, we analyzed their membrane expression in dissociated cortical cultures. We selected mutants representing all three glycosylation groups: A7 and A8 (immature glycosylation and no Cl^−^ extrusion activity), B8 and C1 (mature glycosylation and high Cl^−^ extrusion activity), and C11 (intermediate glycosylation and no Cl^−^ extrusion activity). To visualize KCC2 expression at the neuronal plasma membrane, cortical cultures were transfected with the corresponding KCC2 expression constructs, and FLAG-ir was assessed using live-cell surface immunolabeling protocol (Chamma et al., 2013; Friedel et al., 2017). This protocol consists of two steps (Figure 5A): first, revealing FLAG-tags of the KCC2 molecules present in the plasma membrane of non-permeabilized living cells (“Extracellular” in Figure 5B; and second, detecting FLAG-tags in KCC2 molecules in both the plasma membrane and intracellular compartments of permeabilized PFA-fixed cells “Total” in Figure 5B). The ratio of the two signals serves as a measure of the KCC2 plasmalemmal abundance.

Consistent with their low Cl^−^ efflux and immature glycosylation pattern, the A7 and A8 mutants exhibited much lower membrane abundance compared to WT KCC2 (Figure 5C, A7: 0.03 ± 0.01, *p* = 0.004; A8: 0.16 ± 0.04, *p* = 0.004, one-sample Wilcoxon test). In contrast, B8 mutant was presented in the plasma membrane at a level three times higher than WT KCC2 (Figure 5C, B8: 2.96 ± 1.16, *p* = 0.027, one-sample Wilcoxon test) correlating with its high Cl^−^ efflux and mature glycosylation pattern. C1 and C11 mutants showed no difference in either the membrane abundance (Figure 5C) or glycosylation patterns (Figure 4) compared to WT KCC2. Given that C1 exhibited high Cl^−^ efflux while C11 showed no detectable Cl^−^ extrusion activity, these data suggest that the mechanisms regulating C11 trafficking to the plasma membrane and K-Cl cotransporter activity may function independently.

### 3.6 Somatodendritic chloride gradient measured in the cultured cortical neurons

**Figure 6:**
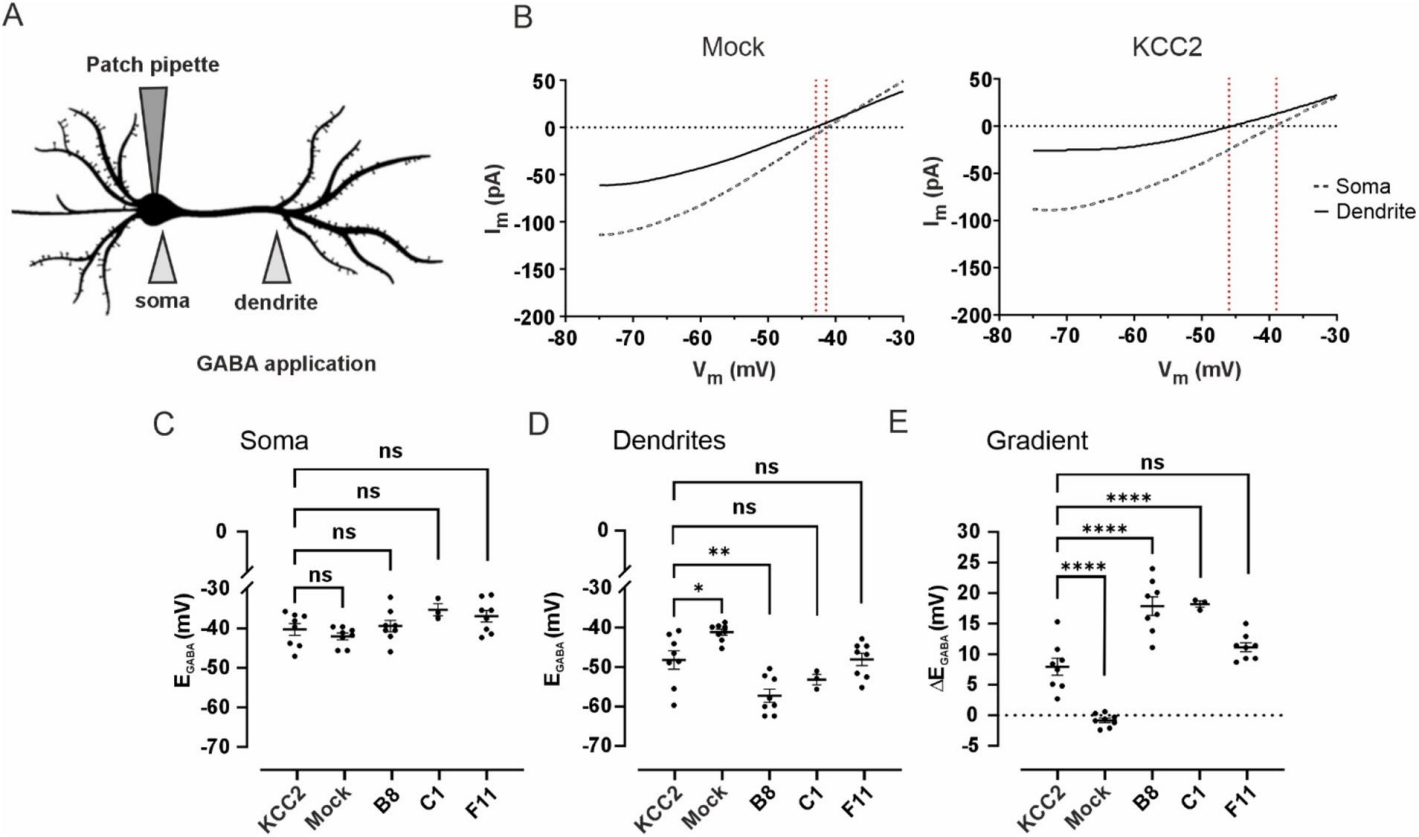
**(A)** Schematic drawing illustrating of the experimental setup for assessing changes in dendritic chloride extrusion. GABA was locally applied at the soma and at the primary dendrite of a transfected neuron recorded in whole-cell voltage clamp mode with a pipette containing 30 mM of Cl^−^. **(B)** Mean voltage ramp trace during local application of GABA at the soma (black dashed line) and the dendrite (black solid line) for Mock- (left panel) and KCC2- (right panel) transfected neurons. Red dashed lines mark an estimation of the GABA_A_ reversal potential at the specific location. **(C)** E_GABA_ values at the soma of neurons transfected with pcDNA3 (Mock), KCC2, and the CTD mutants. Dots represent individual experiments, the horizontal line is drawn at the mean, error bars are SEM. ANOVA with Bonferroni post-hoc test, *p < 0.05, **p < 0.01, ***p < 0.001, ****p < 0.0001, ns – not significant. **(D)** E_GABA_ values at the dendrite of neurons transfected with pcDNA3 (Mock), KCC2, and the CTD mutants. The data presentation and statistical tests are the same as in **(C)**. **(E)** Somatodendritic E_GABA_ gradient (ΔE_GABA_) in neurons transfected with pcDNA3 (Mock), KCC2, and the CTD mutants. The data presentation and statistical tests are the same as in **(C)**.

KCC2-mediated neuronal Cl^−^ efflux is known to be sufficient for maintaining the somatodendritic E_GABA_ gradient (ΔE_GABA_) under whole-cell clamp conditions with a constant somatic Cl^−^ load (Jarolimek et al., 1999; Khirug et al., 2005). To assess the transport activity of KCC2-CTD mutants with the mature (B8, C1) and intermediate (F11) glycosylation patterns, we measured their ΔE_GABA_ in immature DIV 3 dissociated cortical cultures, when the impact of the endogenous KCC2 is negligible (Khirug et al., 2005) (Figure 6).

Mock-transfected neurons revealed no significant ΔE_GABA_: −0.8 ± 0.4 mV, with somatic E_GABA_ −42.0 ± 0.9 mV and dendritic E_GABA_ −41.2 ± 0.8 mV (Figures 6B–E). Transient transfection (24 h) of WT KCC2 resulted in a robust ΔE_GABA_ of 8.0 ± 1.4 mV, with somatic and dendritic E_GABA_ values of −40.3 ± 1.5 mV and −48.2 ± 2.3 mV, respectively. Neurons transfected with the F11 mutant (intermediate glycosylation pattern) exhibited ΔE_GABA_ = 11.1 ± 0.7 mV (somatic E_GABA_ − 36.9 ± 1.5 mV and dendritic E_GABA_ −48.1 ± 1.6 mV), though the difference from WT KCC2 ΔE_GABA_ was not statistically significant. A much steeper ΔE_GABA_ = 17.9 ± 1.5 mV was detected in neurons transfected with the B8 mutant (mature glycosylation pattern) with the somatic and dendritic E_GABA_ −39.4 ± 1.5 mV and −57.2 ± 1.7 mV, respectively. Similarly to B8, neurons transfected with C1 mutant exhibited ΔE_GABA_ of 17.9 ± 0.5 mV, with somatic and dendritic E_GABA_ of −35.8 ± 1.5 mV and −53.7 ± 1.4 mV, respectively.

This experiment corroborates the glycosylation and membrane expression data for the B8 and C1 mutants. Specifically, the significantly increased surface expression of B8 compared to WT KCC2 (Figure 5C) corresponds to the enhanced somatodendritic ΔE_GABA_ gradient (Figure 6E). The fact that WT KCC2 and the C1 mutant exhibit similar membrane expression levels but different Cl^−^ extrusion capacities suggests that the C1 mutation induces a CTD conformation that promotes enhanced kinetics of Cl^−^ extrusion.

## 4 Discussion

Reduction of KCC2 activity and consequent increase in [Cl^−^]_i_ are implicated in numerous neurological conditions, which makes KCC2 a potential drug target (McMoneagle et al., 2024; Tang, 2020). Enhancing Cl^−^ extrusion by activating KCC2 has demonstrated promising outcomes in preclinical trials using mouse models of epilepsy (Jarvis et al., 2023). However, effective drug design requires understanding of the mechanisms governing KCC2 activity. Here we report generation of a library of KCC2 mutants with single 5-aa insertions in the distal regulatory region of KCC2-CTD. Our findings reveal novel mutations that lead to accelerated KCC2-mediated Cl^−^ extrusion under Cl^−^ loading conditions as well as mutations that render KCC2 inactive.

We performed the initial screening of the mutants by analyzing their expression pattern in cultured neurons. The functional presentation of KCC2 on the neuronal membrane is essential for its activity, thus we expected that an aberrant expression of a mutant might correlate with its altered activity. Indeed, out of ten mutants with the aberrant expression that were selected based on immunocytochemical analysis, five (A7, B1, C10, C11, and F7) did not show any detectable VU-sensitive activity in the fluorescent Cl^−^ efflux assay, one (A8) had strongly diminished activity, two (B8 and C1) had strongly enhanced activity, and only two (E4 and F11) had activity similar to WT KCC2 (Figures 3D,E).

KCC2 exists as a dimer in domain-swapping conformation (Chi X. et al., 2021; Xie et al., 2020). By design, mutagenesis in our study was restricted to the outer CTD lobe (Figures 1D, 2B–D), avoiding the previously described scissor helix and β1-β5 strands required for KCC2 dimerization (Chi G. et al., 2021; Zhang et al., 2021), thus we do not expect that these mutations directly interfere with dimerization. The maturation process of KCC2 protein includes glycosylation at specific sites within the third extracellular loop (Agez et al., 2017). KCC2 expression in the plasma membrane is known to correlate with the transporter glycosylation pattern (Hartmann and Nothwang, 2015). Although the mutations reported in this study were limited to the KCC2-CTD and could not directly alter KCC2 glycosylation sites, faulty processing of KCC2 mutants or their inability to form dimers may lead to retention in the endoplasmic reticulum ER (Trombetta and Parodi, 2003), resulting in an immature glycosylation pattern (Kok et al., 2024). Alterations in protein conformation, disruption of ER exit signals, and interference with ER chaperones and regulatory proteins are all known to affect protein exit from the ER and, consequently, its abundance in the plasma membrane (Anelli and Panina-Bordignon, 2019). For example, mutating the C-terminal di-acidic motif has been shown to prevent NKCC2 from exiting the ER and reaching the plasma membrane, causing it to accumulate in the ER (Bakhos-Douaihy et al., 2022).

The regulation of KCC2 surface expression involves multiple regulatory sites within the KCC2-CTD, including di-leucine motif (Zhao et al., 2008), S940 phosphorylation site (Lee et al., 2007), and a hydrophobic tetrapeptide sequence (VMLV) (Tang, 2016). Interestingly, none of the mutants exhibiting an immature glycosylation pattern (A7, A8, B1, and C10) had insertions proximal to these sites. However, all these insertions, except A7, were located within structural elements such as α-helices or β-strands described recently (Zhang et al., 2021). This suggests that the poor membrane expression of these mutants is likely due to their ER retention caused by protein folding failures.

Loss-of function mutations in KCC2-CTD can result in either a reduced amount of the cotransporter in the plasma membrane or in impaired chloride extrusion without altering its membrane expression. For example, there are indications that phosphorylation status at T906/T1007 might directly modulate intrinsic transport activity of KCC2 (Moore et al., 2018). Similarly, E50_Q93del and M415V mutations associated with epilepsy of infancy with migrating focal seizures suppress Cl^−^ extrusion without affecting KCC2 cell surface expression (Saitsu et al., 2016). Previous studies have also shown that even single-amino acid substitutions (L675A and R1049C) could strongly affect the KCC2 kinetic activity without substantial changes in their plasma membrane expression (Döding et al., 2012; Kahle et al., 2014).

Our western blot data showed that, in most cases, the glycosylation pattern of the CTD mutants corresponded to their Cl^−^ extrusion activity. For example, inactive mutants A7, B1, and C10 did not display the fully glycosylated form on the western blot, while mutants with high Cl^−^ extrusion activity, such as B8 and C1, exhibited a prominent glycosylated band (Figures 3D,E 4B). Interestingly, the C11 mutant displayed a mature glycosylation pattern but did not demonstrate any detectable VU-sensitive Cl^−^ efflux in the Cl^−^ extrusion assay. The live-cell labeling assay conducted in neurons confirmed that both C11 and C1 mutants exhibited membrane abundance similar to WT KCC2 (Figure 5B). One possible explanation for the inactivity of the C11 mutant could be that KCC2 distribution in the plasma membrane might also influence its functional state. Two previous studies highlighted a special role of lipid rafts in regulating KCC2 activity (Watanabe et al., 2009; Hartmann et al., 2009). Another possibility is that KCC2 activity can be controlled independently of its membrane expression.

While numerous loss-of-function KCC2 mutations have been described previously both in humans and rodents (McMoneagle et al., 2024), gain-of-function mutations are far less common. To the best of our knowledge, two hyperactive KCC2 mutants have been described so far, both affecting KCC2 phosphorylation status. The first is the double mutant T906A/T1007A that renders impossible the phosphorylation of KCC2 at these sites (Rinehart et al., 2009). Cultured neurons ectopically expressing the T906A/T1007A mutant exhibited strongly hyperpolarized E_GABA_ values compared to WT neurons (Inoue et al., 2012; Moore et al., 2019). The second is a phosphomimetic S937D, which showed an increased KCC2 activity in HEK293 cells and in neurons from transgenic T934A/S937D mice (Radulovic et al., 2023; Weber et al., 2014). In the current study, we present two more mutants with enhanced Cl^−^ extrusion capacity, B8 and C1. Both mutants have insertions located outside known structural and phosphorylation motifs (Table 2 and Supplementary Figure 1) in regions surrounding highly conserved β6-α5-β7 structural elements (Figure 2A). B8 insertion site is moderately conserved among KCC2 paralogs, indicating that B8 mutation might disrupt an unidentified regulatory element. In contrast, C1 site shows low homology with other KCCs, and it appears that the specific 5-amino acid insertion in C1 (CGRTG at residue 817) renders the mutated KCC2 hyperactive. Supporting this, the F11 mutant, which contains a VRPHP insertion at residue 816, just one amino acid upstream of C1, exhibits normal Cl^−^ extrusion kinetics.

In conclusion, we utilized the Mu transposon-based mutagenesis system to generate a mutant library targeting KCC2-CTD, which contained 221 mutants within ~300 aa distal CTD region and 67 mutants in the proximal 3’UTR. We analyzed mutants with ectopic neuronal expression to explore how specific mutations affect Cl^−^ extrusion activity and membrane expression. We identified both gain-of-function and loss-of-function mutations and confirmed that the high cotransporter activity overall correlated with the mature glycosylation status. Nevertheless, one loss-of-function mutant (C11) demonstrated a mature glycosylation pattern and no cotransporter activity suggesting that the KCC2 kinetic activity can be regulated in parallel to a membrane expression.

Given the diverse regulatory functions of KCC2-CTD, our CTD insertion library promotes identification of novel motifs modulating various aspects of KCC2 activity. Further systematic analysis of the positions of the CTD mutations could provide valuable insights into the mechanisms governing KCC2 regulation and potentially discover new targets for therapeutic intervention in neurological disorders.

## Data Availability

The raw data supporting the conclusions of this article will be made available by the authors, without undue reservation.
