# Erratum: Uncovering novel KCC2 regulatory motifs through a comprehensive transposon-based mutant library

**DOI:** 10.3389/fnmol.2025.1576660

**Published:** 2025-02-25

**Authors:** Frontiers Production Office

**Affiliations:** Frontiers Media SA, Lausanne, Switzerland

**Keywords:** KCC2, SLC12A5, GABA, chloride homeostasis, Mu transposon mutagenesis, KCC2-CTD mutations

Due to a production error, there was a mistake in the published legend of Figure 4, resulting in the incorrect use and duplication of the legend for Figure 3. The corrected Figure 4 and its legend appear below.

**Figure 4:**
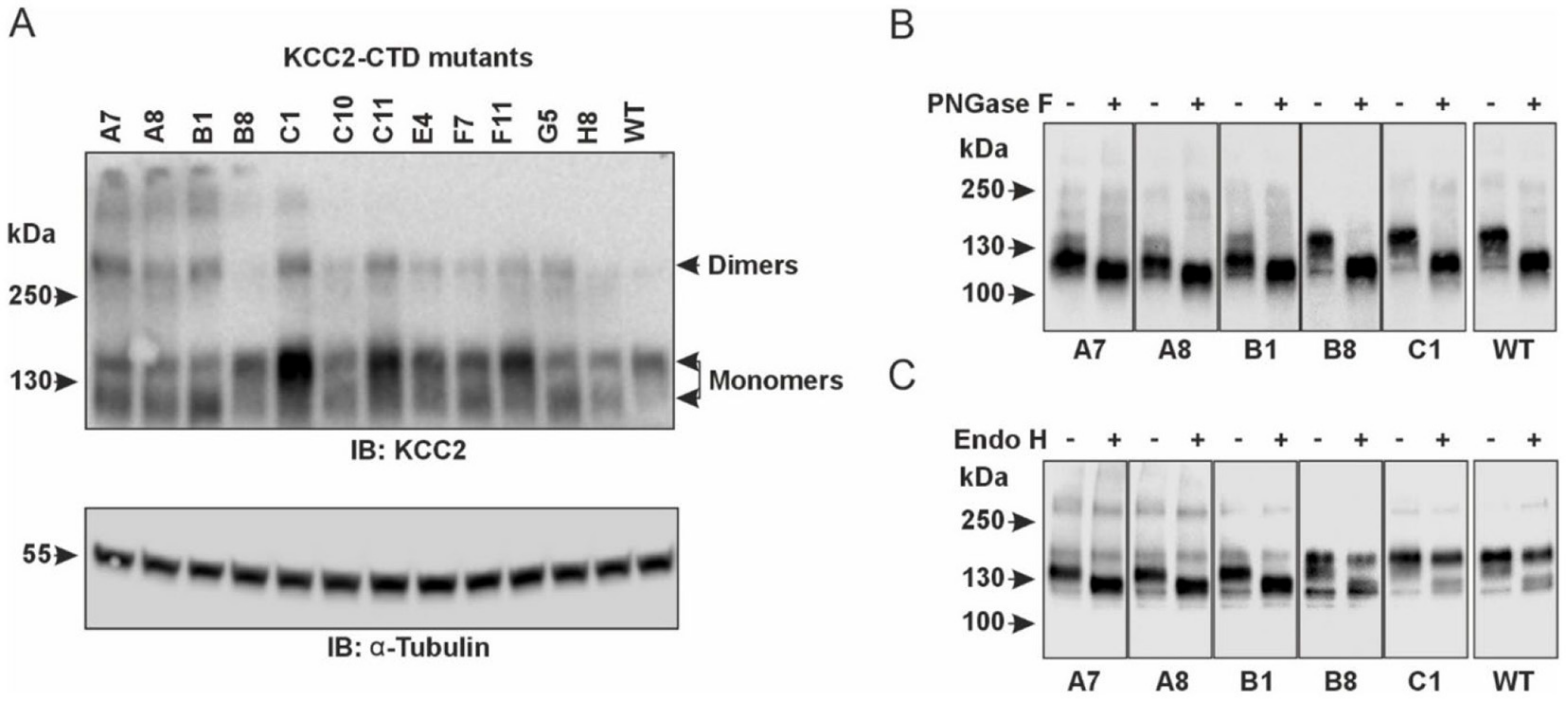
Glycosylation patterns of the KCC2-CTD mutants. **(A)** Top panel: Western blot analysis of KCC2 polypeptides in the total protein lysates of HEK293 cells expressing KCC2-CTD mutants, using a KCC2 antibody recognizing the C-terminal epitope. Two bands around 130-kDa corresponding to the putative glycosylated forms of monomeric KCC2 are observed. Bottom panel: To ensure equal amounts of total proteins loaded on SDS–PAGE, blots were analyzed with the antibody recognizing α-Tubulin. **(B)** PNGase F treatment removes all N-linked oligosaccharides from the KCC2 polypeptides, thus shifting the KCC2 bands to their predicted unglycosylated molecular weight of 123.6-kDa. **(C)** Endo H treatment shifts down the 125-kDa band corresponding to the A7, A8, and B1 KCC2 mutants, which contain mainly ER-added high-mannose glycans but leaves intact the 140-kDa KCC2 band corresponding to the B8 and C1 mutants, which contain mainly Golgi-added complex glycans.

The publisher apologizes for this mistake.

The original article has been updated.

