# Supplementary material for: Uncovering novel KCC2 regulatory motifs through a comprehensive transposon-based mutant library
Source: Front Mol Neurosci. 2025 Jan 15;17:1505722. doi: 10.3389/fnmol.2024.1505722 (PMC11774852; doi:10.3389/fnmol.2024.1505722)
Supplement: Supplementary file 1 [file Supplementary_material.docx]

**Supplementary material**


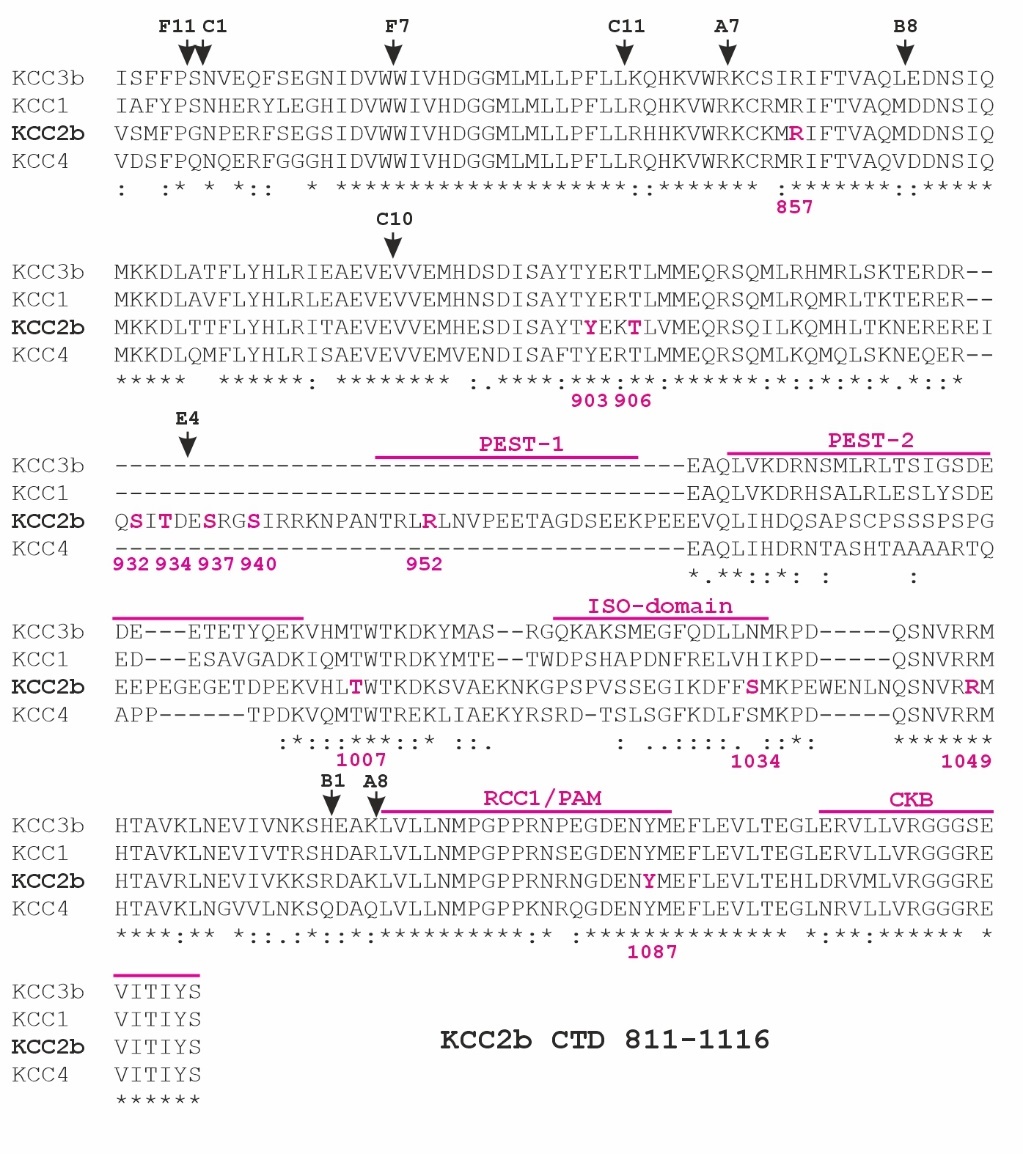


Suppl. Fig. 1. Protein sequence alignment of the distal CTD region for rat KCC2 orthologs. Previously characterized phosphorylation sites (Y903, T906, S932, T934, S937, S940, T1007, S1034, Y1087), regulatory domains (ISO and PEST), and binding motifs of KCC2-interacting partners (SH3/VAV2, RCC1/PAM, and CKB) are shown. Three arginine residues previously found to be mutated in patients with epilepsy of infancy with migrating focal seizures (R857) (Saito et al., 207), febrile seizures (R952) (Puskarjov et al., 2014), and idiopathic generalized epilepsy (R1049) (Kahle et al., 2014) are shown.


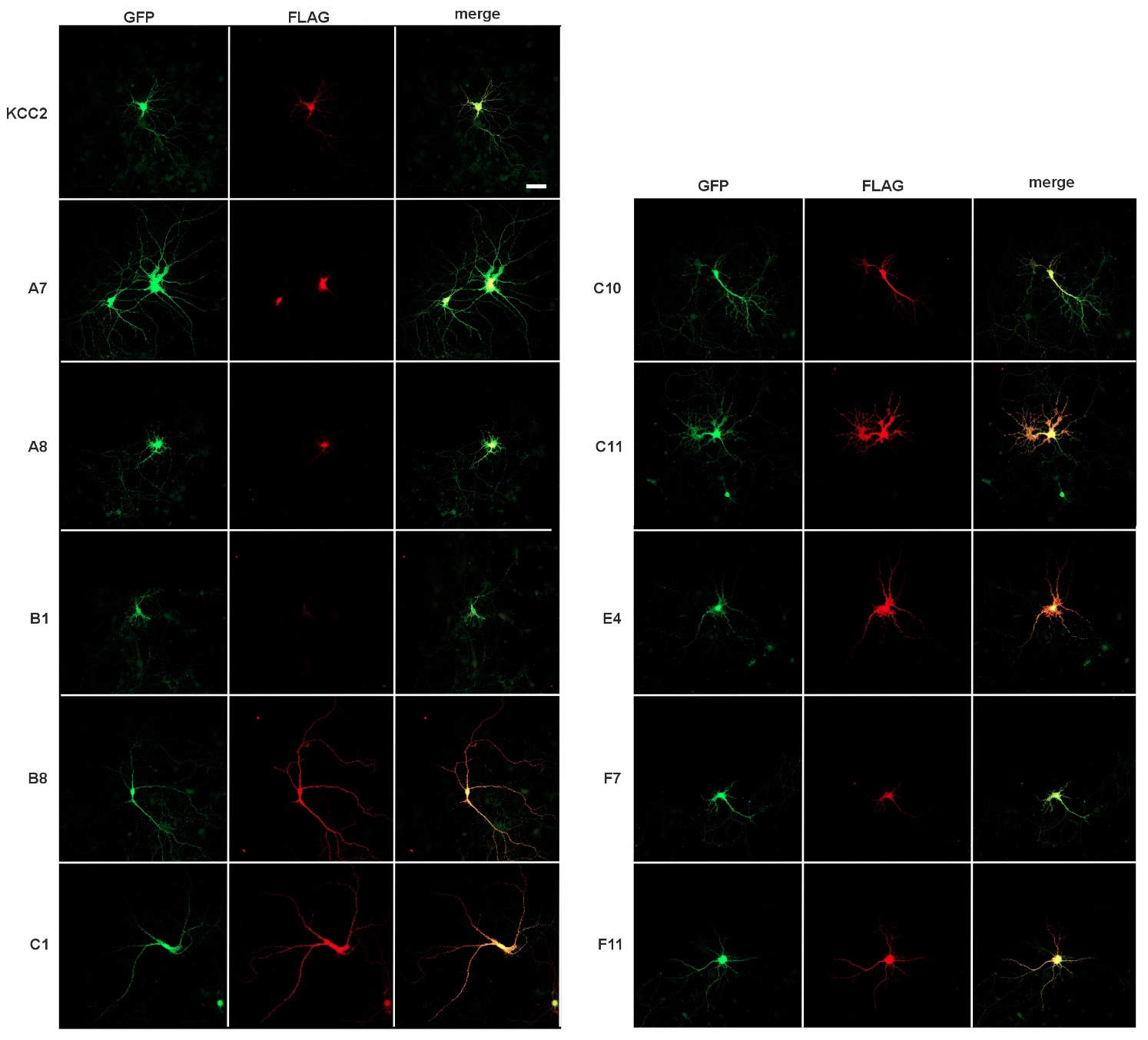


Suppl. Fig. 2. Representative images of cortical neurons expressing WT KCC2 and selected KCC2-CTD mutants. Panels show GFP expression, FLAG-ir corresponding to the mutant expression, and the merge. Sale bar is 50 μm


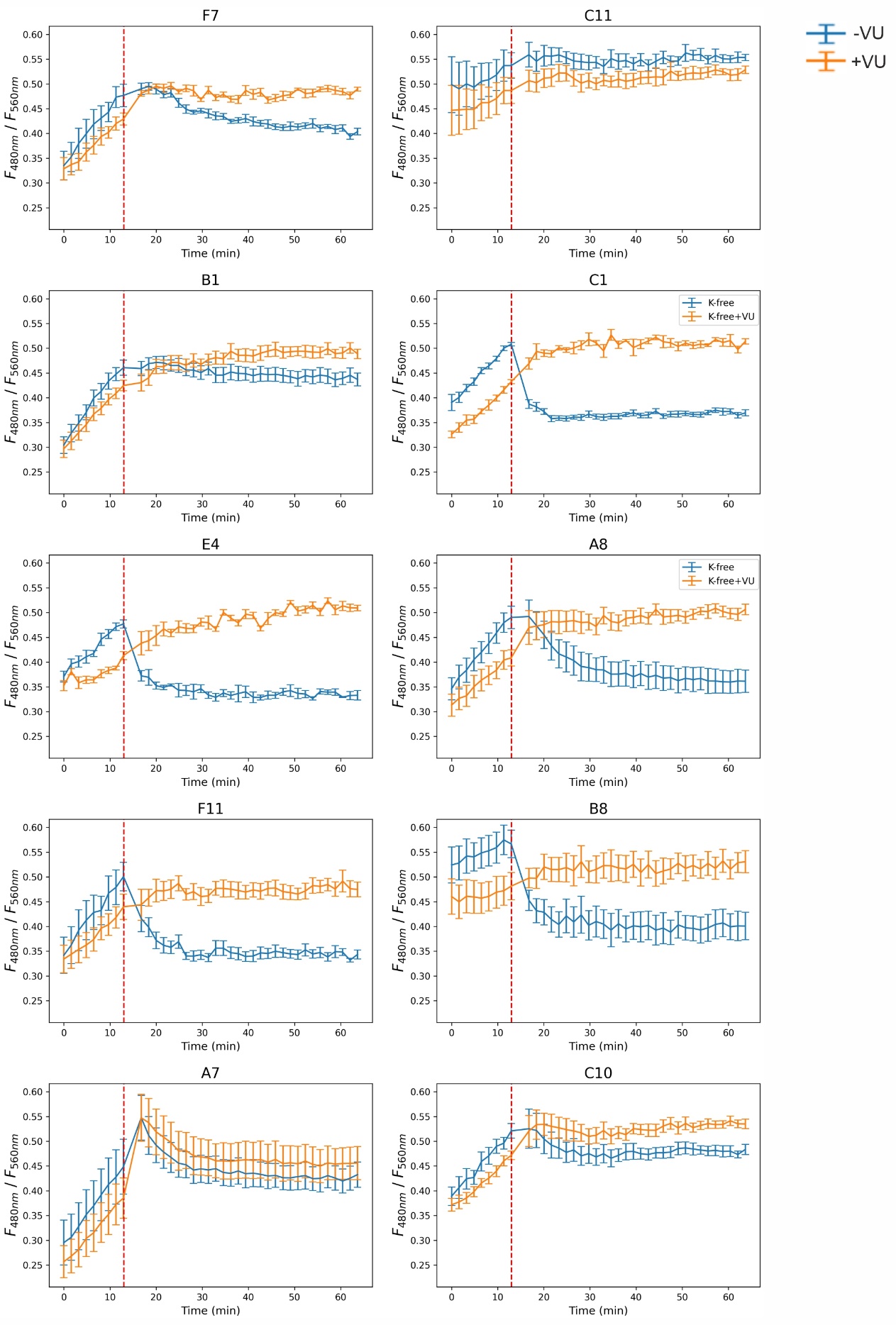


Suppl. Fig. 3. F_480_/F_560_ ratio over time for the cells transfected with the KCC2-CTD mutants in the absence or presence of VU. The start of the extrusion step is marked with a vertical red dashed line.
